# Signaling Transduction of ABA, ROS, and Ca^2+^ in Plant Stomatal Closure in Response to Drought

**DOI:** 10.3390/ijms232314824

**Published:** 2022-11-26

**Authors:** Hui Liu, Songbo Song, Hui Zhang, Yanhua Li, Liangjie Niu, Jinghua Zhang, Wei Wang

**Affiliations:** College of Life Sciences, National Key Laboratory of Wheat and Maize Crop Science, Henan Agricultural University, Zhengzhou 450046, China

**Keywords:** stomatal closure, drought, water-deficit stress, ABA, ROS, Ca^2+^

## Abstract

Drought is a global threat that affects agricultural production. Plants have evolved several adaptive strategies to cope with drought. Stomata are essential structures for plants to control water status and photosynthesis rate. Stomatal closure is an efficient way for plants to reduce water loss and improve survivability under drought conditions. The opening and closure of stomata depend on the turgor pressure in guard cells. Three key signaling molecules, including abscisic acid (ABA), reactive oxygen species (ROS), and calcium ion (Ca^2+^), play pivotal roles in controlling stomatal closure. Plants sense the water-deficit signal mainly via leaves and roots. On the one hand, ABA is actively synthesized in root and leaf vascular tissues and transported to guard cells. On the other hand, the roots sense the water-deficit signal and synthesize CLAVATA3/EMBRYO-SURROUNDING REGION RELATED 25 (CLE25) peptide, which is transported to the guard cells to promote ABA synthesis. ABA is perceived by pyrabactin resistance (PYR)/PYR1-like (PYL)/regulatory components of ABA receptor (RCAR) receptors, which inactivate PP2C, resulting in activating the protein kinases SnRK2s. Many proteins regulating stomatal closure are activated by SnRK2s via protein phosphorylation. ABA-activated SnRK2s promote apoplastic ROS production outside of guard cells and transportation into the guard cells. The apoplastic H_2_O_2_ can be directly sensed by a receptor kinase, HYDROGEN PEROXIDE-INDUCED CA^2+^ INCREASES1 (HPCA1), which induces activation of Ca^2+^ channels in the cytomembrane of guard cells, and triggers an increase in Ca^2+^ in the cytoplasm of guard cells, resulting in stomatal closure. In this review, we focused on discussing the signaling transduction of ABA, ROS, and Ca^2+^ in controlling stomatal closure in response to drought. Many critical genes are identified to have a function in stomatal closure under drought conditions. The identified genes in the process can serve as candidate genes for genetic engineering to improve drought resistance in crops. The review summarizes the recent advances and provides new insights into the signaling regulation of stomatal closure in response to water-deficit stress and new clues on the improvement of drought resistance in crops.

## 1. Introduction

A long period of drought reduces soil moisture and causes water-deficit stress, which profoundly represses plant growth and reduces crop productivity [1]. With the intensification of climate change, drought has become more and more frequent worldwide. It is urgent and necessary to improve crops’ drought resistance, which is defined as the ability of plants to cope with drought [1,2]. Plants have evolved three strategies to adapt to drought, including drought escape, drought tolerance, and drought avoidance [2,3,4]. Drought escape is referred to the acceleration of a plant’s life cycle before stress affects its survival. Drought tolerance is referred to maintaining growth with low water content over the drought period by osmotic adjustment, reactive oxygen species (ROS) scavenging, and activation of stress-related genes. Drought avoidance is referred to as reducing water loss by fast stomatal closure and long-term growth inhibition until the arrival of the next rain [2,4,5]. Among those ways, stomatal closure is recognized as one of the most effective strategies to improve drought resistance. The rapid stomatal response helps plants survive in an environment of water deficiency by reducing water loss [3,6,7]. Stomata are specialized structures composed of a pair of guard cells in leaves and have a history of at least 4 million years of evolution [8]. Stomata control the exchange of carbon dioxide (CO_2_) and water between leaves and the atmosphere [9,10]. The stomatal movement is important for plants to regulate photosynthesis and transpiration. The opening and closure of stomata are mainly determined by variations in the turgor pressure of guard cells, which are regulated through complex molecular signaling pathways [11].

ABA, ROS, and Ca^2+^ are important signal molecules involved in many abiotic stresses, such as water-deficit stress, cold stress, heat stress, and salt stress [6,7,10,12,13]. Stomatal movement is important for plants to cope with abiotic stresses [6]. When facing water-deficit stress stimuli, plants rapidly close their stomata to prevent water loss [1]. The closure of stomata is regulated by many components, such as receptors, protein kinases, transporters, and ion channels. Most of them are regulated by ABA, ROS, and Ca^2+^-mediated cellular signaling [10]. ABA is a key determinative for stomatal closure under drought conditions [14]. Water-deficit signal rapidly induces stomatal closure, mainly depending on ABA. ABA synthesis and accumulation in guard cells are induced by water-deficit stress [14]. Meanwhile, ABA is transported from other tissues to guard cells [15]. ABA promotes stomatal closure by regulating downstream signaling components.

ROS are well-known harmful oxidants that can damage proteins, lipids, and nucleic acids of cells when excessive. However, numerous studies show that ROS are also important signaling molecules that regulate plant growth, development, and stress responses [12,13,16,17]. ROS include singlet oxygen (^1^O_2_), superoxide (O_2·_^−^), hydroxyl radical (·OH), and hydrogen peroxide (H_2_O_2_) [16,18,19]. ROS play a key role in controlling stomatal closure in response to water-deficit stress [14,18,20]. Exogenous application of H_2_O_2_ on the epidermal layer of leaves induces stomatal closure [21]. Under drought conditions, ROS production is enhanced in different compartments, including cell membranes, chloroplasts, and peroxisomes [22]. Many critical factors contributing to the generation of ROS have been characterized [16]. The increased ROS can cause protein activity alteration by oxidative post-translational modifications [18,23]. Several proteins directly regulated by ROS have been identified and shown a crucial function in controlling stomatal closure under drought conditions [16,18]. Recent advance identifies the receptor of H_2_O_2_, which makes great progress in understanding ROS signaling in stomatal closure [24]. In addition, ROS are shown to participate in ABA-mediated stomatal closure [14]. The production and accumulation of apoplastic ROS depend on ABA signaling [24], suggesting both ABA and ROS are important for stomatal closure.

Ca^2+^ is an important second messenger which acts in the process of stomatal closure [6]. Stomatal movement correlates with the concentration of guard cell cytosolic Ca^2+^. Increased concentration of cytosolic Ca^2+^ induces stomatal closure [24,25,26]. Water deficit stress rapidly causes an increase in the concentration of cytosolic Ca^2+^. The increased Ca^2+^ activates calcium-dependent protein kinases (CPKs), calcineurin-B-like proteins (CBLs), and CBL-interacting protein kinases (CIPKs) [10]. Many CPKs, CBLs, and CIPKs have been identified to function in stomatal closure in response to water-deficit stress [10]. In addition, Ca^2+^-induced stomatal closure is regulated by ABA and ROS, which can increase the concentration of cytosolic Ca^2+^ in guard cells. Several ABA signaling components are regulated by Ca^2+^-mediated signaling [10].

In this review, ABA, ROS, and Ca^2+^-mediated signaling in stomatal closure in response to drought are summarized. The three molecules form complex signaling pathways to regulate stomatal closure. Many critical proteins that regulate stomatal closure are listed. We summarize the recent advances and establish a network to integrate the regulatory pathways of stomatal closure by ABA, ROS, and Ca^2+^ in response to drought. The review will be valuable for the understanding of stomatal movement in coping with stresses.

## 2. ABA Signaling-Mediated Stomatal Closure in Response to Drought

Water-deficit stress triggers the synthesis and accumulation of ABA in roots, which is transported to shoots to modulate stomatal closure [15]. ABA is also synthesized in the shoot, primarily in the vascular tissues [27,28]. ATP binding cassette (ABC) family proteins, such as ABCG25 and ABCG40, have been identified as ABA transporters. ABCG25 functions as an ABA exporter from the vasculature, and ABCG40 functions as an ABA importer to guard cells, suggesting an active transport of ABA from the vasculature to the guard cells [15,29,30]. In addition, plant roots sense the water-deficit stress and generate a signal peptide, CLAVATA3/EMBRYO-SURROUNDING REGION RELATED 25 (CLE25), which is transported to the outside of guard cells through vascular tissues. CLE25 activates expression of *NINE-CIS EPOXYCAROTENOID DIOXYGENASE 3 (NCED3*), encoding a key enzyme for ABA synthesis, via BARELY ANY MERISTEM 1 (BAM1) and BAM3 receptors kinases, and thus promotes stomatal closure by modulating ABA synthesis in guard cells [31]. The signaling molecules for activation of *CLE25* in roots and the target proteins directly phosphorylated by BAM1 and BAM3 to promote *NCED3* expression are still unknown. Thus, a drought-induced increase in ABA in guard cells arises from two parts; one part is directly synthesized in guard cells, and another part is transported from the roots and vascular tissues of leaves.

**Figure 1:**
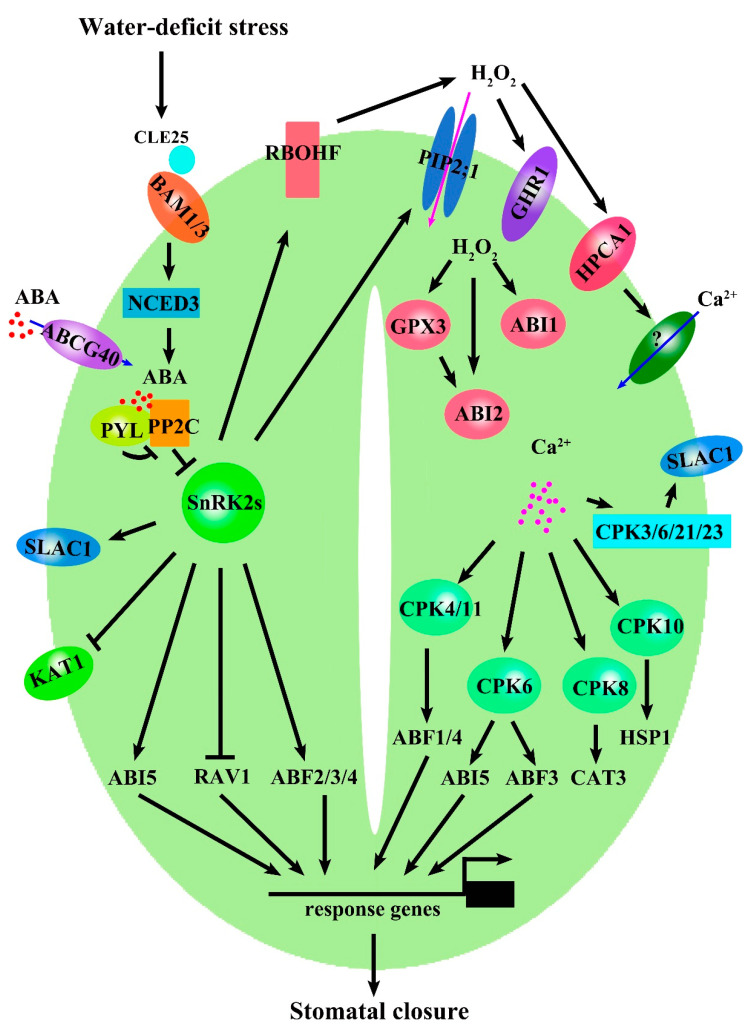
Water-deficit stress signaling is initiated by the increase in ABA levels in guard cells. ABA is perceived by receptors PYR/PYL/RCAR, which inactivate PP2Cs, resulting in the activation of SnRK2s. ABA-activated SnRK2s promote stomatal closure by modulation of proteins, including SLAC1, RBOHF, KAT, and PIP21. Water-deficit stress can also trigger ROS and Ca^2+^ accumulation. ABA promotes ROS generation outside of guard cells by RBOHF and promotes H_2_O_2_ transport into guard cells by PIP2;1. The apoplastic H_2_O_2_ is perceived by the receptor HPCA1 and induces activation of unknown Ca^2+^ channels, resulting in increase in Ca^2+^ in the cytoplasm of guard cells. ABA plays a central role in this process, whereas ROS and Ca^2+^ promote ABA-induced stomatal closure. For detailed explanation, please see Section 5.

The accumulated ABA is perceived by the receptors pyrabactin resistance (PYR)/PYR1-like (PYL)/regulatory components of ABA receptor (RCAR). The binding affinity of ABA-PYR/PYL/RCAR is regulated by clade A type 2C phosphatases (PP2Cs), which are regarded as ABA co-receptors [10]. PP2Cs play negative roles in the ABA signaling by inhibition of downstream targets, such as SNF1-related protein kinase 2.2 (SnRK2.2), SnRK2.3, and SnRK2.6/Open Stomata 1 (OST1). It has been well demonstrated that the module constituted by PYR/PYL/RCAR, PP2Cs, and SnRK2s plays a core role in the early response to ABA. SnRK2s function to induce stomatal closure in response to drought by phosphorylating multiple proteins, including the S-type efflux anion channel SLOW ANION CHANNEL-ASSOCIATED 1 (SLAC1), the inward K^+^ channel POTASSIUM CHANNEL IN ARABIDOPSIS THALIANA 1 (KAT1). *SLAC1* encodes a plasma membrane-localized anion channel, which is preferentially expressed in guard cells [32]. SLAC1 is essential for the efflux of Cl^-^ and NO_3_^-^ from guard cells. Stomatal closure requires SLAC1 in response to various environmental signals such as ABA, CO_2,_ and H_2_O_2_. The *slac1* mutant shows higher stomatal conductance and more water loss than the wild type. The difference in water loss is not a result of variation in the number of stomata between the wild type and the *slac1* mutant [32]. POTASSIUM CHANNEL IN ARABIDOPSIS THALIANA 1 (KAT1) encodes an inward K^+^ channel localized on the plasma membrane in guard cells [33]. KAT1 functions in K^+^ uptake into guard cells during light-induced stomatal opening [34]. When plants encounter drought, ABA-activated SnRK2.6 (OST1) can phosphorylate KAT1 to inhibit K^+^ influx into guard cells, which results in stomatal closure [35] (Figure 1).

Many transcription factors involved in stomatal closure are identified as direct targets of SnRK2s (Figure 1). Protein phosphorylation by SnRK2s is required for the transcription factor to regulate stomatal closure. SnRK2s can phosphorylate several bZIP transcription factors, including ABF2/AREB1, ABF3, ABF4/AREB2, and ABI5, which specially bind to a conserved cis-element known as the ABA-responsive element (ABRE, PyACGTCG/TC), and regulate stomatal closure [36,37,38,39]. The APETALA2/Ethylene Responsive Factor (AP2/ERF) transcription factor RAV1 is another target of SnRK2s [40,41]. In vitro kinase assays show that SnRK2.2, SnRK2.3, and SnRK2.6 can phosphorylate RAV1 and negatively regulate its transcriptional activity. RAV1 can directly bind to the ABI3, ABI4, and ABI5 promoters to repress their expressions in ABA signaling [40]. Under drought conditions, *RAV1*-overexpressing transgenic lines show higher water loss than the wild type. Overexpression of *RAV1* inhibits the ABA-induced stomatal closure [42]. RAV1 is a negative regulator for ABA-mediated stomatal closure. ABA-activated SnRK2s negatively regulate RAV1 activity via protein phosphorylation (Figure 1). The networks based on ABA signaling rapidly control stomatal closure in response to drought. Enhancing ABA signaling helps plants improve drought resistance and survive in a water-deficit stress environment.

## 3. ROS Signaling-Mediated Stomatal Closure in Response to Drought

RESPIRATORY BURST OXIDASE HOMOLOG PROTEIN D (RBOHD) and RBOHF are responsible for the production of apoplastic ROS out of guard cells. Decreased ROS levels by double mutation of RBOHD and RBOHF impair ABA-induced stomatal closure [20]. Water-deficit stress induces the production of apoplastic ROS in guard cells depending on ABA signaling. ABA triggers the production of ROS in guard cells mainly by modulating the activity of RBOHD and RBOHF oxidases [20]. ABA-activated SnRK2.6/OST1 can phosphorylate RBOHF to regulate its activity in vitro [43]. Whether RBOHD is phosphorylated by SnRK2s remains unknown.

RBOHD and RBOHF have NADPH or NADH binding sites, which can transfer electrons to apoplastic O_2_ to produce O_2_·^−^. The O_2_·^−^ can be converted to H_2_O_2_ by superoxide dismutases (SODs). Identification of ROS sensors is a key challenge for ROS signaling in plants. Recent advances have made great progress in the identification of ROS receptors. The apoplastic H_2_O_2_ can be directly sensed by a membrane-localized leucine-rich repeat receptor kinase (LRR-RK), HYDROGEN PEROXIDE-INDUCED Ca^2+^ INCREASES1 (HPCA1) [24]. H_2_O_2_ activates HPCA1 by covalent modification of its extracellular cysteine residues, resulting in autophosphorylation of HPCA1. Loss of function of *HPCA1* impairs Ca^2+^ increase and stomatal closure induced by the application of extracellular H_2_O_2_. H_2_O_2_-activated HPCA1 induces increases of Ca^2+^ in guard cells and promotes stomatal closure [24]. *GUARD CELL HYDROGEN PEROXIDE-RESISTANT1* (*GHR1*) encodes a receptor-like kinase localized on the plasma membrane. Loss of function of GHR1 causes the repression of H_2_O_2_-induced stomatal closure by inhibition of the S-type anion channel SLOW ANION CHANNEL-ASSOCIATED1 (SLAC1) and Ca^2+^ channel [21]. GHR1 physically interacts with, phosphorylates, and activates SLAC1 when coexpressed in *Xenopus laevis* oocytes. However, a lack of kinase in GHR1 can also activate SLAC1 in oocytes, suggesting phosphorylation of GHR1 is not required for the activation of SLAC1 [44]. GHR1 activates SLAC1 might by other unknown mechanisms such as changing conformation. GHR1 might phosphorylate other substrates to regulate the Ca^2+^ channel or directly phosphorylate the Ca^2+^ channel [21].

The apoplastic H_2_O_2_ can be transferred to the cytoplasm by an aquaporin PLASMA MEMBRANE INTRINSIC PROTEIN 2;1 (PIP2;1), which can be phosphorylated by ABA-activated SnRK2.6/OST1. Mutation of PIP2;1 shows a defect in ABA-induced ROS production and stomatal closure [45]. ABA treatment promotes ROS accumulation in the cytoplasm, chloroplasts, nucleus, and endomembrane structures. Chloroplasts and peroxisomes are major sources of ROS in the inner of guard cells [14]. Drought-induced ROS accumulation in the outside of guard cells mainly depends on ABA-mediated RBOHs, whereas, that in the inner of guard cells mainly arises from the ABA-mediated transporter PIP2;1, and the synthesis in chloroplasts and peroxisomes.

ROS in the cytosol regulate protein activity by oxidative modification [23]. The ROS scavenger GLUTATHIONE PEROXIDASE3 (GPX3) is identified as one target of ROS. Compared with the wild type, the *atgpx3* mutant causes impaired ABA- and H_2_O_2_-induced stomatal closure and faster water loss, resulting in the reduction of drought resistance, suggesting ATGPX3 plays an important role in ABA-mediated stomatal closure under drought conditions [46]. In addition, ATGPX3 can physically interact with the 2C-type protein phosphatase ABA INSENSITIVE2 (ABI2). Both ATGPX3 and ABI2 are found to be oxidized by H_2_O_2_. The phosphatase activity of ABI2 in vitro is significantly reduced by the addition of oxidized ATGPX3 [46]. ATGPX3 might regulate stomatal closure by modulation of ABI2-mediated ABA signaling in response to drought. ABI1 is another PP2C protein that exerts a negative function in ABA signaling. H_2_O_2_ reversibly inhibits ABI1 activity in vitro [47]. Thus, H_2_O_2_ in the cytosol may inhibit the activity of ATGPX3, ABI2, and ABI1 to regulate ABA signaling and stomatal closure (Figure 1).

As for ROS, water-deficit stress induces apoplastic ROS production outside of guard cells via ABA-activated RBOHF, which contributes to the generation of O_2_·^−^. The O_2_·^−^ can be converted to H_2_O_2_, which is perceived by the receptor HPCA1 and activates an unknown Ca^2+^ channel to trigger an increase in Ca^2+^ concentration and promote stomatal closure [24]. Meanwhile, apoplastic H_2_O_2_ is transported to guard cells via PIP2;1, which is regulated by ABA-activated SnRK2.6/OST1 [45]. In the inner of guard cells, drought induces ROS accumulation, mainly in the chloroplasts and peroxisomes [14]. The receptor of H_2_O_2_ in the cytoplasm remains unknown. Several proteins, including ATGPX3, ABI2, and ABI1, are regulated by H_2_O_2_ to modulate stomatal closure [46,47] (Figure 1).

## 4. Ca^2+^ Signaling-Mediated Stomatal Closure in Response to Drought

CALCIUM-DEPENDENT PROTEIN KINASE 6 (CPK6) positively regulates stomatal closure and drought resistance via phosphorylating ABF3 and ABI5 transcription factors. CPK6-mediated phosphorylation of ABF3 and ABI5 enhances their transcriptional activities [48]. CPK6 can also directly phosphorylate and activate SLAC1 [49]. CPK8 functions in Ca^2+^-mediated plant responses to drought. The *cpk8* mutant is more sensitive to drought compared with wild-type plants. CATALASE3 (CAT3) is identified as a CPK8-interacting protein. CPK8 can phosphorylate CAT3 at Ser-261 and regulate its activity. Both *cpk8* and *cat3* plants show lower catalase activity and higher accumulation of H_2_O_2_ compared with wild-type plants. The *cat3* mutant displays a similar drought-sensitive phenotype as the *cpk8* mutant [50]. Loss of function of *CPK10* results in the sensitive phenotype to drought, while overexpression of *CPK10* causes enhanced drought resistance. HSP1 is identified as a CPK10-interacting protein. The *hsp1* mutant shows a similar sensitive phenotype to drought as the *cpk10* mutant. ABA- and Ca^2+^ -mediated repression of inward K^+^ currents in stomatal guard cells are impaired in the *cpk10* and *hsp1* mutants. CPK10 possibly regulates ABA- and Ca^2+^- mediated stomatal movements by interacting with HSP1 [51]. Loss of function of *CPK4* and *CPK11* results in the repression of ABA-induced stomatal closure. The CPK4 and CPK11 can both phosphorylate the transcription factors ABF1 and ABF4 in vitro [52]. CPK4 and CPK11 positively regulate stomatal closure via phosphorylation of ABA-responsive factors ABF1 and ABF4. Mutation of *CPK23* results in reduced stomatal apertures [53]. CPK23 and CPK21 are identified as interacting partners of SALC1 by split YFP-based protein–protein interaction assays. SLAC1 can be phosphorylated and activated by CPK23 and CPK21 [54]. In addition to CPK6, CPK21, and CPK23, CPK3 is also reported to activate SLAC1 by direct phosphorylation [55]. CPK33 is identified to play an important role in the process of Ca2^+^ -induced stomatal closure. The *cpk33* mutant is also impaired in H_2_O_2_-induced stomatal closure [56]. These results suggest that Ca^2+^ has a crucial role in regulating stomatal movement under drought conditions.

During drought, ABA induces stomatal closure through Ca^2+^-dependent and Ca^2+^-independent signaling pathways. In the absence of Ca^2+^, a subset of stomata can also close, indicating that Ca^2+^ is not essential for ABA-induced stomatal closure. If the Ca^2+^ signal is activated by ABA, the stomata close faster, suggesting Ca^2+^ accelerates the phase of stomatal closure [57]. The protein kinase OST1 is a critical factor in the process of ABA-activated Ca^2+^ signal. Defects of OST1 repress Ca^2+^ signal and prevent ABA-induced stomatal closure. Elevation of the cytosolic Ca^2+^ concentration results in rapid activation of SLAC1 and SLAH3 anion channels. During stomatal closure, ABA activates the Ca^2+^ signal by OST1. These Ca^2+^ signals are likely to activate Ca^2+^-dependent protein kinases, which enhance the activity of SLAC1 and SLAH3 and accelerate stomatal closure [57]. The accumulation of Ca^2+^ concentration in the cytoplasm induced by ABA can be sensed by the protein kinases such as CPK, CaM, and CIPK. Many ABA core signaling factors, such as ABF1, ABF3, ABF4, and ABI5, are direct targets of CPKs [48,52]. In addition, H_2_O_2_ induces increase of cytosolic-free Ca^2+^ concentration and causes stomatal closure [58]. H_2_O_2_ triggers an influx of Ca^2+^ ions, indicating there is signal transduction from ROS to Ca^2+^. By using forward genetic screens based on Ca^2+^ imaging, HPCA1 is identified as an essential factor in connecting ROS and Ca^2+^ [24]. The oxidative modification of HPCA1 results in its autophosphorylation, which induces activation of Ca^2+^ channels and mediates Ca^2+^ influx into guard cells, resulting in stomatal closure [24].

## 5. Signaling Transduction of ABA, ROS, and Ca^2+^ in Regulating Stomatal Closure

Plants sense water-deficit stress and close stomata via a complex signaling pathway. ABA is the core signal molecule in the process of drought-induced stomatal closure. When plants encounter water-deficit stress, ABA is rapidly synthesized in the roots and shoots and transported to guard cells by ABA transporters. At the same time, roots also generate a signal peptide CLE25 and transmit it to the outside of guard cells via vascular tissues. CLE25 activates the expression of a core ABA synthesis enzyme gene *NCED3* by receptor kinases BAM1 and BAM3. The accumulated ABA in guard cells is perceived by the receptors PYR/PYL/RCAR, which deactivate the PP2Cs, resulting in the activation of SnRK2s. Many proteins regulating stomatal closure, such as SLAC1, KAT1, RBOHF, and PIP2;1, are direct targets of SnRK2s. SnRK2s promote the production of apoplastic O_2_·^−^ of guard cells by activation of RBOHF. The O_2_·^−^ can be converted to H_2_O_2_, which is perceived by the receptor kinase HPCA1. HPCA1 senses and transduces the H_2_O_2_ signal into the guard cell via Ca^2+^. H_2_O_2_ triggers an increase in Ca^2+^ concentration in the guard cells’ cytoplasm. In addition, the apoplastic H_2_O_2_ can be transferred to the cytoplasm by an aquaporin PIP2;1, which is regulated by ABA-mediated SnRK2s. H_2_O_2_ in the cytoplasm regulates stomatal closure via the modulation of proteins, including ATGPX3, ABI2, and ABI1. Thus, we illustrate a signal pathway that regulates stomatal closure in response to water-deficit stress via ABA, ROS, and Ca^2+^ (Figure 1).

ABA plays a central role in this process, whereas ROS and Ca^2+^ promote ABA-induced stomatal closure. The ABA and Ca^2+^ signaling pathways have been well studied in the past decades; however, the target proteins of ROS in stomatal closure are poorly understood (Table 1). We suggest that future studies should focus on identifying new proteins that are directly regulated by ROS. Particularly, searching for ROS sensors is the most important event for understanding ROS signaling. The elucidation of the regulatory network in stomatal closure would be helpful for improving drought resistance in crops.

## 6. Comparison of Fast Reply of Stomata and Prolonged Changes via Protein Synthesis in Response to Drought

When facing drought, plants rapidly close the stomata to adapt to the water-deficit environment [1]. In this process, ABA is critical for a fast reply to stress [69]. ROS and Ca^2+^ enhance the action of ABA-mediated stomatal closure [20,57]. The early response to water-deficit stress is regulated mainly via phosphorylation and oxidative modification of proteins. After a long period of drought stress, ABA activates many stress-responsive proteins synthesis via transcription factors to make plants obtain drought resistance [70]. The stress-related proteins are involved in accumulations of sugar and proline and increase of antioxidant enzyme activity, which contribute to improving drought resistance. Many transcription factors are identified to regulate stress-related protein synthesis, such as AP2/ERF, bHLH, bZIP, DREB, HD-Zip, MYB, NAC, WRKY. [70]. For example, the transcription factor gene *AtbHLH112* is induced by drought and ABA, and overexpression of *AtbHLH112* improves drought resistance by increasing the expression of *P5CS* genes and reducing the expression of *P5CDH* and *ProDH* genes to increase proline levels. AtbHLH112 also increases the expression of *POD* and *SOD* genes to improve reactive oxygen species (ROS) scavenging ability [71]. The prolonged changes in plants are important to adapt to drought conditions. The stomatal closure prevents water loss and helps cope with early stimuli from water-deficit stress; however, long-time closure of stomata would limit plant growth. Thus, the synthesis of stress-related proteins makes plants obtain stress resistance to drought via increasing the contents of sugar and proline or enhancing the activity of the antioxidant enzyme. Plants have evolved diverse strategies to adapt to drought environments.

## 7. Drought Resistance Improvement: From Arabidopsis to Crops

Water is critical for plant survival; however, the availability of water is predicted to drop by 50% due to climate change in 2050 [1]. To survive in a water-limited environment, the plant can reduce water loss by rapidly closing its stomata. Engineering the stomatal movement signaling helps to improve drought resistance. Genetic engineering of ABA signaling components can promote stomatal closure and improve drought resistance. Many genes are identified to promote stomatal closure and improve drought resistance in crops (Table 1). For example, overexpression of a wheat ABA receptor gene *TaPYL4* reduces stomatal aperture size and water loss, resulting in improved drought resistance [59]. Overexpression of *TaABL1*, which is an ABA-responsive element-binding protein (AREB), promotes stomatal closure and improves drought resistance [60]. Overexpression of *ZmPIF1*, *ZmPIF3*, *ZmOST1*, and *ZmHK9* can improve drought resistance by ABA-mediated regulation of stomatal closure [61,62,63,64]. By genome-wide association study, ZmSRO1d is identified as a critical gene regulating drought resistance. Overexpression of *ZmSRO1d* enhances drought resistance by activating the mono-ADP-ribosyltransferase activity of ZmRBOHC, which increases ROS levels in guard cells and promotes stomatal closure [65]. Two calcium-dependent proteins, ZmCPK35 and ZmCPK37, enhance maize drought tolerance by activating anion channel ZmSLAC1 in guard cells and promoting stomatal closure [66]. Wheat *TaCIPK23*-overexpression shows a higher survival rate under drought conditions by modulation of ABA signaling and induction of stomatal closure [67]. The rice CDPK gene *OsCPK9* can improve drought resistance by enhancing stomatal closure [68]. The strategy can be used to increase water-use efficiency in other crops by genetic engineering of stomatal movement regulators because of the high conservation of signaling pathways [72,73].

The pathway that regulates stomatal closure under drought conditions has been well studied in *Arabidopsis*; however, whether and how the multiple signals, including ABA, Ca^2+^, and ROS, control stomatal closure in crops remains largely unknown (Table 1). The studies that improve crop drought resistance by manipulation of the stomatal closure pathway are also few. Focus on the regulation of stomatal closure can help improve crop performance and increase agricultural yields under drought conditions [72,73]. Thus, identifying genes involved in stomatal closure and revealing their molecular mechanism are important for drought resistance improvement in crops.

## 8. Conclusions

Stomata are important structures in plant development and stress adaptation. The stomatal aperture is regulated by multiple signal molecules. ABA, ROS, and Ca^2+^ are key molecules in determining stomatal closure under drought conditions. The regulatory networks based on ABA, ROS, and Ca^2+^-mediated signaling are critical for stomatal closure (Figure 1). The increases in ABA, ROS, and Ca^2+^ in guard cells are important for the fast reply of stomata to water-deficit stress. The ABA signaling module PYL-PP2C-SnRK2s functions to rapidly close stomata by activation of several key proteins, including SLAC1, KAT1, RBOHF, and PIP2;1. ROS and Ca^2+^ acting as second messengers enhance ABA signaling. ABA, ROS, and Ca^2+^ coordinate to regulate stomatal closure in response to drought.

We should pay attention to the fact that stomatal closure, in addition to reducing water loss under drought conditions, limits CO_2_ intake and photosynthesis. Fast stomatal closure helps reduce water loss and improves drought resistance, but it also represses plant growth. This is impairing the progress on genetic engineering of stomatal closure to improve crop drought resistance. Therefore, those genes which can improve drought resistance by reducing stomatal conductance while not restricting photosynthesis should be given more attention. Improving drought resistance without a reduction in CO_2_ uptake and photosynthesis can occur by partially reducing stomatal conductance and increasing water use efficiency under drought conditions [74]. For example, Arabidopsis expressing the poplar ABA receptor gene *RCAR10* reduces stomatal conductance and shows enhanced water use efficiency and drought resistance compared with the wild type [75]. Similarly, overexpression of a wheat ABA receptor gene *TaPYL4* results in drought resistance and water-saving traits, which are a consequence of reduced stomatal conductance and increased photosynthesis [59]. By modulating ABA receptors, drought resistance is improved by increasing water use efficiency without penalizing plant growth, although the stomatal conductance is reduced [59,74,75]. Thus, improvement of crop drought resistance can be achieved by engineering the stomata to maximize water use efficiency. Water use efficiency should be paid more attention to in future studies.

## Figures and Tables

**Table 1:** Genes involved in promoting stomatal closure in response to water-deficit stress.

Signaling Pathway	Genes	Species	Reference
ABA	*CLE25*	Arabidopsis	[31]
	*BAM1*	Arabidopsis	[31]
	*BAM3*	Arabidopsis	[31]
	*NCED3*	Arabidopsis	[31]
	*SLAC1*	Arabidopsis	[32]
	*KAT1*	Arabidopsis	[35]
	*ABF2/AREB1*	Arabidopsis	[36,37]
	*ABF3*	Arabidopsis	[37]
	*ABF4/AREB2*	Arabidopsis	[37]
	*RAV1*	Arabidopsis	[42]
	*TaPYL4*	wheat	[59]
	*TaABL1*	wheat	[60]
	*ZmPIF1*	maize	[61]
	*ZmPIF3*	maize	[62]
	*ZmOST1*	maize	[63]
	*ZmHK9*	maize	[64]
ROS	*HPCA1*	Arabidopsis	[24]
	*GHR1*	Arabidopsis	[21,44]
	*ATGPX3*	Arabidopsis	[46]
	*RBOHD*	Arabidopsis	[20]
	*RBOHF*	Arabidopsis	[20]
	*CAT3*	Arabidopsis	[50]
	*PIP2;1*	Arabidopsis	[45]
	*ZmSRO1d*	maize	[65]
	*ZmRBOHC*	maize	[65]
Ca^2+^	*CPK3*	Arabidopsis	[55]
	*CPK4*	Arabidopsis	[52]
	*CPK6*	Arabidopsis	[48]
	*CPK8*	Arabidopsis	[50]
	*CPK10*	Arabidopsis	[51]
	*CPK11*	Arabidopsis	[52]
	*CPK21*	Arabidopsis	[54]
	*CPK23*	Arabidopsis	[53,54]
	*ZmCPK35*	maize	[66]
	*ZmCPK37*	maize	[66]
	*ZmSLAC1*	maize	[66]
	*TaCIPK23*	wheat	[67]
	*OsCPK9*	rice	[68]

## Data Availability

Not applicable.

## References

[1] Gupta A., Rico-Medina A., Caño-Delgado A.I. (2020). The physiology of plant responses to drought. Science.

[2] Skirycz A., Inzé D. (2010). More from less: Plant growth under limited water. Curr. Opin. Biotechnol..

[3] Daszkowska-Golec A., Szarejko I. (2013). Open or close the gate—Stomata action under the control of phytohormones in drought stress conditions. Front. Plant Sci..

[4] Kooyers N.J. (2015). The evolution of drought escape and avoidance in natural herbaceous populations. Plant Sci..

[5] Shohat H., Cheriker H., Kilambi H.V., Illouz Eliaz N., Blum S., Amsellem Z., Tarkowská D., Aharoni A., Eshed Y., Weiss D. (2021). Inhibition of gibberellin accumulation by water deficiency promotes fast and long-term ‘drought avoidance’ responses in tomato. New Phytol..

[6] Agurla S., Gahir S., Munemasa S., Murata Y., Raghavendra A.S. (2018). Mechanism of stomatal closure in plants exposed to drought and cold stress. Adv. Exp. Med. Biol..

[7] Kollist H., Zandalinas S.I., Sengupta S., Nuhkat M., Kangasjärvi J., Mittler R. (2019). Rapid responses to abiotic stress: Priming the landscape for the signal transduction network. Trends Plant Sci..

[8] Chater C.C.C., Caine R.S., Fleming A.J., Gray J.E. (2017). Origins and evolution of stomatal development. Plant Physiol..

[9] Li S., Li X., Wei Z., Liu F. (2020). ABA-mediated modulation of elevated CO(_2_) on stomatal response to drought. Curr. Opin. Plant. Biol..

[10] Gong Z., Xiong L., Shi H., Yang S., Herrera-Estrella L.R., Xu G., Chao D.Y., Li J., Wang P.Y., Qin F. (2020). Plant abiotic stress response and nutrient use efficiency. Sci. China Life Sci..

[11] Dong H., Bai L., Zhang Y., Zhang G., Mao Y., Min L., Xiang F., Qian D., Zhu X., Song C.P. (2018). Modulation of guard cell turgor and drought tolerance by a peroxisomal acetate-malate shunt. Mol. Plant.

[12] Pardo-Hernández M., López-Delacalle M., Rivero R.M. (2020). ROS and NO regulation by melatonin under abiotic stress in plants. Antioxidants.

[13] Hasanuzzaman M., Bhuyan M., Zulfiqar F., Raza A., Mohsin S.M., Mahmud J.A., Fujita M., Fotopoulos V. (2020). Reactive Oxygen Species and antioxidant defense in plants under abiotic stress: Revisiting the crucial role of a universal defense regulator. Antioxidants.

[14] Postiglione A.E., Muday G.K. (2020). The role of ROS homeostasis in ABA-induced guard cell signaling. Front. Plant Sci..

[15] Anfang M., Shani E. (2021). Transport mechanisms of plant hormones. Curr. Opin. Plant Biol..

[16] Qi J., Song C.-P., Wang B., Zhou J., Kangasjärvi J., Zhu J.-K., Gong Z. (2018). Reactive oxygen species signaling and stomatal movement in plant responses to drought stress and pathogen attack. J. Integr. Plant Biol..

[17] Yang S., Yu Q., Zhang Y., Jia Y., Wan S., Kong X., Ding Z. (2018). ROS: The fine-tuner of plant stem cell fate. Trends Plant Sci..

[18] Singh R., Parihar P., Singh S., Mishra R.K., Singh V.P., Prasad S.M. (2017). Reactive oxygen species signaling and stomatal movement: Current updates and future perspectives. Redox Biol..

[19] Sierla M., Waszczak C., Vahisalu T., Kangasjärvi J. (2016). Reactive Oxygen Species in the regulation of stomatal movements. Plant Physiol..

[20] Kwak J.M., Mori I.C., Pei Z.M., Leonhardt N., Torres M.A., Dangl J.L., Bloom R.E., Bodde S., Jones J.D., Schroeder J.I. (2003). NADPH oxidase AtrbohD and AtrbohF genes function in ROS-dependent ABA signaling in Arabidopsis. EMBO J..

[21] Hua D., Wang C., He J., Liao H., Duan Y., Zhu Z., Guo Y., Chen Z., Gong Z. (2012). A plasma membrane receptor kinase, GHR1, mediates abscisic acid- and hydrogen peroxide-regulated stomatal movement in Arabidopsis. Plant Cell.

[22] Cruz de Carvalho M.H. (2008). Drought stress and reactive oxygen species: Production, scavenging and signaling. Plant Signal Behav..

[23] Waszczak C., Akter S., Jacques S., Huang J., Messens J., Van Breusegem F. (2015). Oxidative post-translational modifications of cysteine residues in plant signal transduction. J. Exp. Bot..

[24] Wu F., Chi Y., Jiang Z., Xu Y., Xie L., Huang F., Wan D., Ni J., Yuan F., Wu X. (2020). Hydrogen peroxide sensor HPCA1 is an LRR receptor kinase in Arabidopsis. Nature.

[25] McAinsh M.R., Clayton H., Mansfield T.A., Hetherington A.M. (1996). Changes in stomatal behavior and guard cell cytosolic free calcium in response to oxidative stress. Plant Physiol..

[26] Pei Z.M., Murata Y., Benning G., Thomine S., Klüsener B., Allen G.J., Grill E., Schroeder J.I. (2000). Calcium channels activated by hydrogen peroxide mediate abscisic acid signalling in guard cells. Nature.

[27] Bauer H., Ache P., Lautner S., Fromm J., Hartung W., Al-Rasheid K.A., Sonnewald S., Sonnewald U., Kneitz S., Lachmann N. (2013). The stomatal response to reduced relative humidity requires guard cell-autonomous ABA synthesis. Curr. Biol..

[28] Ma Y., Cao J., He J., Chen Q., Li X., Yang Y. (2018). Molecular mechanism for the regulation of ABA homeostasis during plant development and stress responses. Int. J. Mol. Sci..

[29] Kang J., Hwang J.U., Lee M., Kim Y.Y., Assmann S.M., Martinoia E., Lee Y. (2010). PDR-type ABC transporter mediates cellular uptake of the phytohormone abscisic acid. Proc. Natl. Acad. Sci. USA.

[30] Kuromori T., Miyaji T., Yabuuchi H., Shimizu H., Sugimoto E., Kamiya A., Moriyama Y., Shinozaki K. (2010). ABC transporter AtABCG25 is involved in abscisic acid transport and responses. Proc. Natl. Acad. Sci. USA.

[31] Takahashi F., Suzuki T., Osakabe Y., Betsuyaku S., Kondo Y., Dohmae N., Fukuda H., Yamaguchi-Shinozaki K., Shinozaki K. (2018). A small peptide modulates stomatal control via abscisic acid in long-distance signalling. Nature.

[32] Vahisalu T., Kollist H., Wang Y.F., Nishimura N., Chan W.Y., Valerio G., Lamminmaki A., Brosche M., Moldau H., Desikan R. (2008). SLAC1 is required for plant guard cell S-type anion channel function in stomatal signalling. Nature.

[33] Sutter J.U., Campanoni P., Tyrrell M., Blatt M.R. (2006). Selective mobility and sensitivity to SNAREs is exhibited by the Arabidopsis KAT1 K^+^ channel at the plasma membrane. Plant Cell.

[34] Kwak J.M., Murata Y., Baizabal-Aguirre V.M., Merrill J., Wang M., Kemper A., Hawke S.D., Tallman G., Schroeder J.I. (2001). Dominant negative guard cell K+ channel mutants reduce inward-rectifying K+ currents and light-induced stomatal opening in arabidopsis. Plant Physiol..

[35] Sato A., Sato Y., Fukao Y., Fujiwara M., Umezawa T., Shinozaki K., Hibi T., Taniguchi M., Miyake H., Goto D.B. (2009). Threonine at position 306 of the KAT1 potassium channel is essential for channel activity and is a target site for ABA-activated SnRK2/OST1/SnRK2.6 protein kinase. Biochem. J..

[36] Fujita Y., Fujita M., Satoh R., Maruyama K., Parvez M.M., Seki M., Hiratsu K., Ohme-Takagi M., Shinozaki K., Yamaguchi-Shinozaki K. (2005). AREB1 is a transcription activator of novel ABRE-dependent ABA signaling that enhances drought stress tolerance in Arabidopsis. Plant Cell.

[37] Yoshida T., Fujita Y., Sayama H., Kidokoro S., Maruyama K., Mizoi J., Shinozaki K., Yamaguchi-Shinozaki K. (2010). AREB1, AREB2, and ABF3 are master transcription factors that cooperatively regulate ABRE-dependent ABA signaling involved in drought stress tolerance and require ABA for full activation. Plant J..

[38] Kang J.Y., Choi H.I., Im M.Y., Kim S.Y. (2002). Arabidopsis basic leucine zipper proteins that mediate stress-responsive abscisic acid signaling. Plant Cell.

[39] Furihata T., Maruyama K., Fujita Y., Umezawa T., Yoshida R., Shinozaki K., Yamaguchi-Shinozaki K. (2006). Abscisic acid-dependent multisite phosphorylation regulates the activity of a transcription activator AREB1. Proc. Natl. Acad. Sci. USA.

[40] Feng C.Z., Chen Y., Wang C., Kong Y.H., Wu W.H., Chen Y.F. (2014). Arabidopsis RAV1 transcription factor, phosphorylated by SnRK2 kinases, regulates the expression of ABI3, ABI4, and ABI5 during seed germination and early seedling development. Plant J..

[41] Feng J.X., Liu D., Pan Y., Gong W., Ma L.G., Luo J.C., Deng X.W., Zhu Y.X. (2005). An annotation update via cDNA sequence analysis and comprehensive profiling of developmental, hormonal or environmental responsiveness of the Arabidopsis AP2/EREBP transcription factor gene family. Plant Mol. Biol..

[42] Fu M., Kang H.K., Son S.H., Kim S.K., Nam K.H. (2014). A subset of Arabidopsis RAV transcription factors modulates drought and salt stress responses independent of ABA. Plant Cell Physiol..

[43] Sirichandra C., Gu D., Hu H.C., Davanture M., Lee S., Djaoui M., Valot B., Zivy M., Leung J., Merlot S. (2009). Phosphorylation of the Arabidopsis AtrbohF NADPH oxidase by OST1 protein kinase. FEBS Lett..

[44] Sierla M., Horak H., Overmyer K., Waszczak C., Yarmolinsky D., Maierhofer T., Vainonen J.P., Salojarvi J., Denessiouk K., Laanemets K. (2018). The receptor-like pseudokinase GHR1 is required for stomatal closure. Plant Cell.

[45] Grondin A., Rodrigues O., Verdoucq L., Merlot S., Leonhardt N., Maurel C. (2015). Aquaporins contribute to aba-triggered stomatal closure through OST1-mediated phosphorylation. Plant Cell.

[46] Miao Y., Lv D., Wang P., Wang X.C., Chen J., Miao C., Song C.P. (2006). An Arabidopsis glutathione peroxidase functions as both a redox transducer and a scavenger in abscisic acid and drought stress responses. Plant Cell.

[47] Meinhard M., Grill E. (2001). Hydrogen peroxide is a regulator of ABI1, a protein phosphatase 2C from Arabidopsis. FEBS Lett..

[48] Zhang H., Liu D., Yang B., Liu W.Z., Mu B., Song H., Chen B., Li Y., Ren D., Deng H. (2020). Arabidopsis CPK6 positively regulates ABA signaling and drought tolerance through phosphorylating ABA-responsive element-binding factors. J. Exp. Bot..

[49] Brandt B., Brodsky D.E., Xue S., Negi J., Iba K., Kangasjärvi J., Ghassemian M., Stephan A.B., Hu H., Schroeder J.I. (2012). Reconstitution of abscisic acid activation of SLAC1 anion channel by CPK6 and OST1 kinases and branched ABI1 PP2C phosphatase action. Proc. Natl. Acad. Sci. USA.

[50] Zou J.J., Li X.D., Ratnasekera D., Wang C., Liu W.X., Song L.F., Zhang W.Z., Wu W.H. (2015). Arabidopsis CALCIUM-DEPENDENT PROTEIN KINASE8 and CATALASE3 function in abscisic acid-mediated signaling and H_2_O_2_ homeostasis in stomatal guard cells under drought stress. Plant Cell.

[51] Zou J.J., Wei F.J., Wang C., Wu J.J., Ratnasekera D., Liu W.X., Wu W.H. (2010). Arabidopsis calcium-dependent protein kinase CPK10 functions in abscisic acid- and Ca2+-mediated stomatal regulation in response to drought stress. Plant Physiol..

[52] Zhu S.Y., Yu X.C., Wang X.J., Zhao R., Li Y., Fan R.C., Shang Y., Du S.Y., Wang X.F., Wu F.Q. (2007). Two calcium-dependent protein kinases, CPK4 and CPK11, regulate abscisic acid signal transduction in Arabidopsis. Plant Cell.

[53] Ma S.Y., Wu W.H. (2007). AtCPK23 functions in Arabidopsis responses to drought and salt stresses. Plant Mol. Biol..

[54] Geiger D., Scherzer S., Mumm P., Marten I., Ache P., Matschi S., Liese A., Wellmann C., Al-Rasheid K.A., Grill E. (2010). Guard cell anion channel SLAC1 is regulated by CDPK protein kinases with distinct Ca2+ affinities. Proc. Natl. Acad. Sci. USA.

[55] Scherzer S., Maierhofer T., Al-Rasheid K.A., Geiger D., Hedrich R. (2012). Multiple calcium-dependent kinases modulate ABA-activated guard cell anion channels. Mol. Plant.

[56] Wang X., Lv S., Han X., Guan X., Shi X., Kang J., Zhang L., Cao B., Li C., Zhang W. (2019). The calcium-dependent protein kinase CPK33 mediates strigolactone-induced stomatal closure in arabidopsis thaliana. Front. Plant Sci..

[57] Huang S., Waadt R., Nuhkat M., Kollist H., Hedrich R., Roelfsema M.R.G. (2019). Calcium signals in guard cells enhance the efficiency by which abscisic acid triggers stomatal closure. New Phytol..

[58] Price A.H., Taylor A., Ripley S.J., Griffiths A., Trewavas A.J., Knight M.R. (1994). Oxidative signals in tobacco increase cytosolic calcium. Plant Cell.

[59] Mega R., Abe F., Kim J.S., Tsuboi Y., Tanaka K., Kobayashi H., Sakata Y., Hanada K., Tsujimoto H., Kikuchi J. (2019). Tuning water-use efficiency and drought tolerance in wheat using abscisic acid receptors. Nat. Plants.

[60] Xu D.B., Gao S.Q., Ma Y.Z., Xu Z.S., Zhao C.P., Tang Y.M., Li X.Y., Li L.C., Chen Y.F., Chen M. (2014). ABI-like transcription factor gene TaABL1 from wheat improves multiple abiotic stress tolerances in transgenic plants. Funct. Integr. Genom..

[61] Gao Y., Wu M., Zhang M., Jiang W., Ren X., Liang E., Zhang D., Zhang C., Xiao N., Li Y. (2018). A maize phytochrome-interacting factors protein ZmPIF1 enhances drought tolerance by inducing stomatal closure and improves grain yield in Oryza sativa. Plant Biotechnol. J..

[62] Gao Y., Wu M., Zhang M., Jiang W., Liang E., Zhang D., Zhang C., Xiao N., Chen J. (2018). Roles of a maize phytochrome-interacting factors protein ZmPIF3 in regulation of drought stress responses by controlling stomatal closure in transgenic rice without yield penalty. Plant Mol. Biol..

[63] Wu Q., Wang M., Shen J., Chen D., Zheng Y., Zhang W. (2019). ZmOST1 mediates abscisic acid regulation of guard cell ion channels and drought stress responses. J. Integr. Plant Biol..

[64] Wang B., Guo B., Xie X., Yao Y., Peng H., Xie C., Zhang Y., Sun Q., Ni Z. (2012). A novel histidine kinase gene, ZmHK9, mediate drought tolerance through the regulation of stomatal development in Arabidopsis. Gene.

[65] Gao H., Cui J., Liu S., Wang S., Lian Y., Bai Y., Zhu T., Wu H., Wang Y., Yang S. (2022). Natural variations of ZmSRO1d modulate the trade-off between drought resistance and yield by affecting ZmRBOHC-mediated stomatal ROS production in maize. Mol. Plant..

[66] Li X.D., Gao Y.Q., Wu W.H., Chen L.M., Wang Y. (2022). Two calcium-dependent protein kinases enhance maize drought tolerance by activating anion channel ZmSLAC1 in guard cells. Plant Biotechnol. J..

[67] Cui X.Y., Du Y.T., Fu J.D., Yu T.F., Wang C.T., Chen M., Chen J., Ma Y.Z., Xu Z.S. (2018). Wheat CBL-interacting protein kinase 23 positively regulates drought stress and ABA responses. BMC Plant Biol..

[68] Wei S., Hu W., Deng X., Zhang Y., Liu X., Zhao X., Luo Q., Jin Z., Li Y., Zhou S. (2014). A rice calcium-dependent protein kinase OsCPK9 positively regulates drought stress tolerance and spikelet fertility. BMC Plant Biol..

[69] Hsu P.K., Dubeaux G., Takahashi Y., Schroeder J.I. (2021). Signaling mechanisms in abscisic acid-mediated stomatal closure. Plant J..

[70] Hussain Q., Asim M., Zhang R., Khan R., Farooq S., Wu J. (2021). Transcription Factors Interact with ABA through Gene Expression and Signaling Pathways to Mitigate Drought and Salinity Stress. Biomolecules.

[71] Liu Y., Ji X., Nie X., Qu M., Zheng L., Tan Z., Zhao H., Huo L., Liu S., Zhang B. (2015). Arabidopsis AtbHLH112 regulates the expression of genes involved in abiotic stress tolerance by binding to their E-box and GCG-box motifs. New Phytol..

[72] Blum A. (2015). Towards a conceptual ABA ideotype in plant breeding for water limited environments. Funct. Plant. Biol..

[73] Lawlor D.W. (2013). Genetic engineering to improve plant performance under drought: Physiological evaluation of achievements, limitations, and possibilities. J. Exp. Bot..

[74] Yang Z., Liu J., Poree F., Schaeufele R., Helmke H., Frackenpohl J., Lehr S., von Koskull-Döring P., Christmann A., Schnyder H. (2019). Abscisic acid receptors and coreceptors modulate plant water use efficiency and water productivity. Plant Physiol..

[75] Papacek M., Christmann A., Grill E. (2019). Increased water use efficiency and water productivity of arabidopsis by abscisic acid receptors from Populus canescens. Ann. Bot..

